# Super-enhancer-associated LINC00963 promotes metastasis of gastric cancer through epithelial-mesenchymal transition

**DOI:** 10.1371/journal.pone.0332396

**Published:** 2025-09-18

**Authors:** Hong Du, Tingting Xiang, Ying Xia, Yong Jin, Fahua Deng, Wansong Xia, Hongyu Li, Shuqiang Cheng, Bingxue Lan, Sixi Wei, Cunfeng Song, Hai Huang

**Affiliations:** 1 Center for Clinical Laboratories, The Affiliated Hospital of Guizhou Medical University, Guiyang, China; 2 School of Clinical Laboratory Science, Guizhou Medical University, Guiyang, China; 3 Department of Clinical Laboratory, The First Affiliated Hospital of Guizhou University of Traditional Chinese Medicine, Guiyang, China; 4 Division of Gastroenterology and Hepatology, Department of Medicine and Department of Oncology, Sidney Kimmel Comprehensive Cancer Center, Johns Hopkins University School of Medicine, Baltimore, Maryland, United States of America; 5 Department of Clinical Laboratory, The Second People’s Hospital of Guizhou Province, Guiyang, China; 6 School of Electronic Information and Electrical Engineering, Shanghai Jiao Tong University, Shanghai, China; 7 School of Chemical and Environmental Engineering, Shanghai Institute of Technology, Shanghai, China; The Second Affiliated Hospital, Chongqing Medical University, CHINA

## Abstract

Background: In clinical practice, gastric cancer (GC) is a common malignancy with high morbidity. Accumulating research has revealed that lncRNAs are involved in the development and metastasis of tumor tissues in multiple cancers. As reported, *LINC00963*, a typical lncRNA is aberrantly expressed in gastric cancer. However, the underlying mechanisms of super-enhancers mediating remain unclear. Materials and methods: The GC cell line enhancer-super-enhancer data were downloaded and analyzed from the NCBI database (GSE75595). Combined RT-qPCR and Sanger sequencing were employed to identify three variants of *LINC00963* in gastric cell lines and peripheral blood samples from gastric cancer patients. Western blot was used to detect the expression level of epi-thelial-mesenchymal transition (EMT)-related proteins. Transwell assays were applied to assess the cell invasion and migration. A xenograft model was applied to simulate the tumor development process, during which the effect of *LINC00963* on promoting tu-morigenesis were investigated. Results: Analysis of the GC cell line enhanc-er-super-enhancer data revealed a high expression of *LINC00963* driven by su-per-enhancer. The variant 1 and variant 2 of *LINC00963* exhibited high expression in GC cell line and the peripheral blood of gastric cancers. *LINC00963* expression in the GC cell line was reduced after exposure to a low dose of the bromodomain and extraterminal inhibitor JQ1. Down-regulation of *LINC00963* variant 1 resulted in decreased levels of *β*-*catenin* and *ZEB1* proteins, and the protein expression levels of several marker proteins related to EMT, such as *Vimentin*, *N-cadherin* were observed to decrease (Fig 1). Conclusion: This study demonstrated that the super-enhancer-associated *LINC00963* promoting tumor metastasis in gastric cancer through EMT.

## Introduction

In clinical practice, gastric cancer (GC) is a common malignancy with high morbidity. According to the Global Cancer Report, in 2020, GC ranks 5th in amount of new cancer cases (5.6% of 19.3 million), and 4th in cancer-related deaths (7.7% of 10 million) [1]. Invasion and metastasis are the main reasons for the low survival rate and poor prognosis of GC patients [2,3]. At present, surgical treatment is the main treatment for gastric cancer, and intraoperative regional chemotherapy as an adjuvant therapy for advanced gastric cancer can reduce the mortality rate of tumor recurrence or metastasis [4]. As a consequence, it is important to elucidate the molecular mechanisms of GC invasion and metastasis in order to identify effective GC prognostic molecular markers and corresponding therapeutic targets.

A super-enhancer (SE) is a genomic region in mammals, often spanning an average size of up to 30 kb, that comprises multiple enhancers [5]. These enhancers work collectively to recruit core regulatory circuitry transcription factors (TFs), thus becoming an important cause of the mediated transcriptional dysregulation which is observed in human cancers [6,7]. Super-enhancers regulate the abnormal expression of specific genes to promote the malignant process of tumors [8] in pancreatic cancer [9], colorectal cancer [10], Ewing sarcoma [11], and GC [12]. It has also been reported that super-enhancers are more sensitive to drugs, such as JQ1 and THZ1, than typical enhancers [13]. Low doses of JQ1 can interfere with super-enhancer function, thereby inhibiting the abnormal expression of the related downstream target genes, most of which are specific genes that can identify cells. As a result, the occurrence and development of tumors were suppressed [8,14,15]. Enhancer-mediated genes have been reported to promote tissue invasion, such as myocyte enhancer factor 2C (*MEF2C*) [1]. Our previous research has identified several lncRNAs associated with the occurrence, development, and metastasis of gastric cancer [16,17]. We speculate that since SE can modulate specific genes expression to promote the process of tumor development, it is also conceivable that SEs may similarly regulate the lncRNAs expression, resulting in the promotion of malignant progression. However, there have been few reports on super-enhancer-associated genes regulating invasion and migration in GC to date.

This work investigated the function and its underlying mechanisms of a SE-associated gene *LINC00963* in promoting GC progression with in vivo, in vitro experiments and clinical cases. The underlying molecular mechanisms suggested that SE-associated *LINC0096*3 might influence GC invasion and migration through EMT. The findings indicated that *LINC00963* could serve as a potential marker for identifying metastatic progression in GC.

## Materials and methods

Cells cultivation

Gastric cancer cell lines (AGS, MKN45, HGC-27, and MGC803) and an immortalized gastric epithelial cell line (GES-1) were purchased from the National Infrastructure of Cell Line Resource of China (Beijing, China). All cell lines were cultured in RPMI-1640 medium (Gibco, Carlsbad, California, USA) containing 10% fetal bovine serum (Lonza Science SRL, Basel, Switzerland) and 1% penicillin-streptomycin (Invitrogen, Shanghai, China) and incubated under 5% CO2 at 37 ^°^C.

Human GC samples

The peripheral blood of 30 patients with GC and the peripheral blood of 30 healthy individuals at the time of physical examinations were collected 2mL from GC patients and healthy individuals in the Affiliated Hospital of Guizhou Medical University (Gui-zhou, China). The specimen is peripheral venous whole blood containing EDTA antico-agulant. The blood is stored in a $-80\;{\;}^{\circ }\text{C}$ freezer within 6 hours after isolation. The whole blood RNA is extracted and reverse transcribed once a month from the collected speci-mens, and stored in a $-20\;{\;}^{\circ }\text{C}$ freezer for future use. The collection of peripheral blood samples was authorized and approved by the Ethics Committee of the Affiliated Hospital of Guizhou Medical University (2023 [086]). Blood samples are taken with the written consent of the patient. The start and end time for collecting samples for the project is from March 11, 2023 to July 1, 2023.

Super-enhancer data analysis of GC cell lines

The GC cell line enhancer-super-enhancer data is available through GEO under accession number GSE75595 (Accessed in 2019). Super-enhancers were identified using H3K27ac ChIP-seq data from GC cell lines (GSE75595) via the ROSE algorithm [5]. Genes within ±50 kb of super-enhancer peaks were defined as super-enhancer-associated. Gene Ontology (GO) enrichment analysis was conducted using the Kyoto Encyclopedia of Genes and Genomes (KEGG) biological pathway database (http://www.genome.jp/) to describe the super-enhancer target gene function. To predict enhancer-super-enhancer target gene sets, we performed biological pathway enrichment analysis based on the KEGG database, referencing the comprehensive data analysis of excellent enhancers in the previous work reported by Whyte WA and Wang J [5]. The data was accessed on June 27th, 2019. The Cancer Cell Line Encyclopedia (CCLE) database (www.broadinstitute.org/ccle) was used to comprehensively analyze the gene expression of GC cell lines, particularly the LINC00963 gene expression. The data were accessed on January 27, 2020.

RNA extraction and RT- qPCR analysis

Total RNA was extracted from peripheral blood and the GC cell line using the UNlQ-10 column Trizol RNA extraction kit (B511321-0100; Sangon Biotech Co., Ltd., Shanghai, China). The expression of target genes was quantified with RT-qPCR kits (Takara, Cat. DRR820A), normalized to the HPRT housekeeping gene HPRT. Relative gene expression levels were determined using the comparative threshold cycle (2–ΔΔCT) method. The primer sequences of RT-qPCR are listed in the S3 Appendix.

Small interfering RNA (siRNA) transfection and lentivirus vector construction

The full sequences of *LINC00963* (NR 038955.1) and the newly discovered LINC00963-V1 were obtained according to the NCBI database. Specific siRNAs for LINC00963-V1 were designed using siRNA technology and then synthesized by Shanghai Gene Pharma Co., Ltd. (Shanghai, China). The siRNA sequences are listed in the S3 Appendix. Lipofectamine RNAi MAX reagent (Invitrogen, Shanghai, China) was used to transfect siRNA. Cell lines of stable LINC00963 knockdown were established by lentivirus-mediated delivery of LINC00963-specific shRNA (sh-LINC00963). The GM easyTM lentivirus packaging kit (Genomeditech, Shanghai, China) was used to synthesize lentivirus. The vector of letrivirus is the pPLK plasmid sh-LINC00963 (public protein/plasmid library). The shRNA sequences are listed in the S3 Appendix.

Migration and Invasion Assays

Cells transfected with siRNA for 48 h were harvested and suspended in RPMI-1640 medium. A 24-well plate with a cross-well culture insert and an 8-*μ*m pore size membrane was used to analyze migration activity. A total of 50,000 cells were seeded into the upper insertion chamber in 200 *μ*l of serum-free RPMI-1640 medium, then incubated at 37 ^°^C for 18 h after adding 600 *μ*l of RPMI-1640 containing 10% FBS. The invasion test used the above-mentioned modified migration test with a Matrigel-coated cross-well chamber and 8-*μ*m pore size membranes on a 24-well plate (Corning, Corning, NY, USA). A total of 6×10^4^ cells were plated on Matrigel in the upper chamber in 200 *μ*l of serum-free RPMI-1640 medium and 600 *μ*l of RPMI-1640 containing 20% FBS was added to the bottom chamber. After incubating the cells at 37 ^°^C for 24 h, dye and take photos.. After the experiment, they were fixed in methanol and stained with crystal violet solution for photography [18].

Immunoblotting assay

Immunoblotting assay was performed according to our previous studies [17–19]. The cultured cells were lysed in RIPA lysis extraction buffer (Solarbio, Beijing, China). Supplement protease and phosphatase inhibitors (Solarbio) on ice for 30 minutes. Use BCA protein rapid detection kit (Solarbio) to detect protein quantification. Proteins were separated by SDS-PAGE (Solarbio), and then transferred to a 0.45 *μ* m PVDF membrane (EMD Millipore, Darmstadt, Germany). After blocking with TBST containing 5% skim milk for 2 hours, the membrane was incubated overnight with the first antibody. After removing the membrane, it was washed three times in TBST at room temperature for 10 minutes each time, and then incubated with the second antibody for 2 hours at room temperature. After removing the membrane, it was washed three times in TBST at room temperature for 10 minutes each time. After completion, ECL reagent was used for exposure, and the imaging system was a BIO-RAD analysis system. The results were processed using Image J and normalized with GAPDH from the same biological replicate.The following primary antibodies are used and incubated overnight at $4\;{\;}^{\circ }\text{C}$: *GAPDH* (Bioworld, Nanjing, China; 1:5000); *E-cadherin* (ab40772, Abcam, Boston, Massachusetts, USA; 1:1000); *N-cadherin* (ab76011, Abcam; 1:1000); *vimentin* (ET1610-39, Huabio, Hangzhou, China; 1:20000); *MMP7* (ab205525, Abcam; 1:1000); *β*-*catenin* (ab32572, Abcam; 1:400); *ZEB1* (WL03489, Wanleibio, Shenyang, China; 1:2000); *SNAIL* (WL01863, Wanleibio; 1:2000); and horseradish peroxidase-labeled goat anti-rabbit IgG (Multi Sciences, Hangzhou, China; 1:5000) secondary antibody was incubated for 2 hours and analyzed on an exposure instrument under the action of ECL reagent.

In vivo experiments

Female BALB/c nude mice (Age, 5weeks; Weight, 20±3g) were purchased from the SLRC Laboratory Animal Center (Shanghai, China). Subcutaneous injection of 1 × 10^6^ HGC-27 cells from sh-LINC00963 into the right inguinal region of BALB/c nude mice, and injection of sh NC into the left inguinal region of the same nude mouse. A total of 5 mice. The tumor volume and body weight were monitored every 3 days. Tumor volume was calculated by the following equation: volume = (width2 × length)/2. The nude mice were euthanized by cervical dislocation 21 days later, and the tumors were collected, weighed, and stored at –80 ^°^C for follow-up experiments.

All animal experiments were conducted in accordance with the National Institutes of Health Guide for the Care and Use of Laboratory Animals, while strictly adhering to the ARRIVE guidelines 2.0, and received approval from the Animal Research Ethics Committee of Guizhou Medical University (Approval No. 2200732).

Statistical analysis

Statistical analysis was performed using SPSS25.0 software (IBM, SPSS, Armonk, New York, USA). Means, standard deviations, and unpaired Student’s t test results were used to analyze the data. Relative gene expression was performed using the comparative 2 -^ΔΔCt^ or 2^−ΔCt^ method. The graphical data results of all quantified graphs are detailed in the S4 Appendix.

All experiments were performed with at least 3 independent biological replicates. *P* value < 0.05 was considered to be significant.

## Results

*LINC00963 is a super-enhancer-associated gene in GC*

We conducted an analysis of GEO data, specifically utilizing the downloaded GSE75595 dataset [20], for the characterization of super-enhancers in gastric cancer (GC) (Fig 2A). The analysis revealed the identification of 220, 420, 281, 123, and 72 super-enhancers in AGS cells, MKN45 cells, MKN1 cells, KATOIII cells and SNU016 cells, respectively. Notably, super-enhancer target genes in AGS and KATO-III cell lines were found to be involved in the tight junction signaling pathway, while MKN45 and SNU016 cells exhibited super-enhancer-associated genes primarily participating in the adherens junction signal pathway and the SNU016 cell super enhancer genes are enriched in the tight junction pathway (ranked second) and the adhesins junction pathway (ranked fifth) (Fig 2B). The Venn diagram analysis of the predicted target genes by the enhancer-super-enhancer indicated that *ACTN4, KRT80, ZFP36, LINC00963*, and *MIR21* were common to all cell lines studied (The Venn diagram in Fig 2C). Combined with the research basis of the research group, *LINC00963* is selected as the focus of further research.

**Fig 1:**
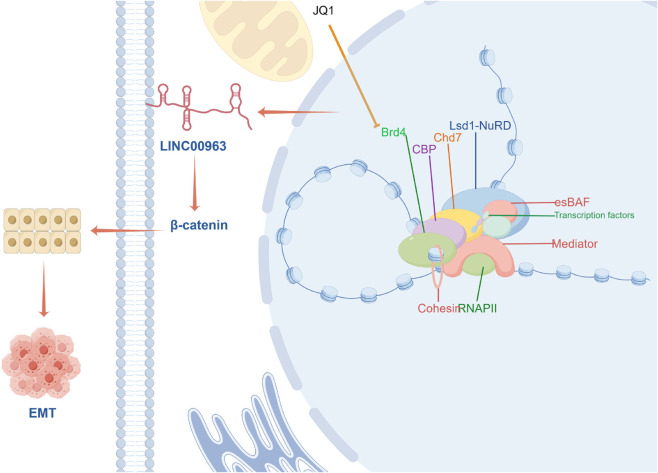
Graphical abstract. Super-enhancer mediated *LINC00963* expression decreased after exposure of GC cell lines to low dose of the bromodomain and extra-terminal inhibitor JQ1. In gastric cancer cells, the super enhancer-mediated upregulation of *LINC00963* results in elevated levels of *β*-*catenin* protein, which facilitate the EMT of gastric cancer cells and consequently enhance invasion and metastasis.

**Fig 2:**
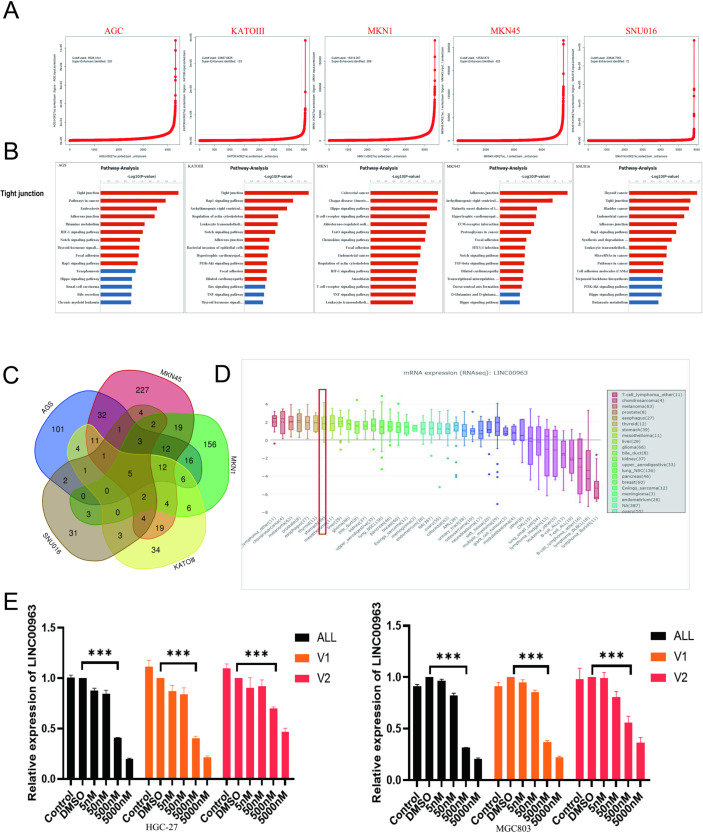
GEO database download data (GSE75595) analysis and identification of gastric cancer cell line super-enhancer and prediction of target genes. (A). H3K27ac to define the active enhancers in the whole cell genome, sorted by H3K27ac enrichment intensity, and found the point with a slope of 1 as the threshold value of normal- and super-enhancers. (B). KEGG analyzed the super-enhancer target gene enrichment signal pathways corresponding to each cell, and it is clear that the main signal regulation pathways in the five cell lines are tight and adherens junctions. (C). A Venn diagram was made for super-enhancer target genes. Five cell lines and five public super-enhancer target genes were found, including *ACTN4, KRT80, ZFP36, LINC00963,* and *MIR21*. (D). CCLE database analysis shows that the *LINC00963* gene is highly expressed in gastric cancer. (E). The inhibitory effect of different doses of the JQ1 inhibitor on HGC-27(left) and MGC803 (right) cell line *LINC00963* different alternative spliceosomes (6 h). *P < 0.05, **P < 0.01, ***P < 0.001.

Using the CCLE database, we investigated the expression of LINC00963 in tumor cell lines derived from the stomach. The results indicated a high expression level in these cell lines (Fig 2D). JQ1 was used to assess its effect on the function of super-enhancer target genes in GC. The inhibitory effect on *BRD4* in tumor cell super-enhancers was predominantly observed after 6 hours of exposure to 500 nM of JQ1 in GC cells. Consequently, GC cell lines were exposed to JQ1 at concentrations of 5, 50, 500, and 5000 nM for 6 hours. The *LINC00963* gene demonstrated a decrease in expression at these concentrations throughout the specified duration. Remarkably, the most significant reduction in the level of *LINC00963* expression in HGC-27 and MGC803 cell lines (Fig 2E) occurred at a JQ1 concentration of 500 nM.

*LINC00963 is over-expressed in GC cell lines and peripheral blood, and it has alternative splice variants*

**Fig 3:**
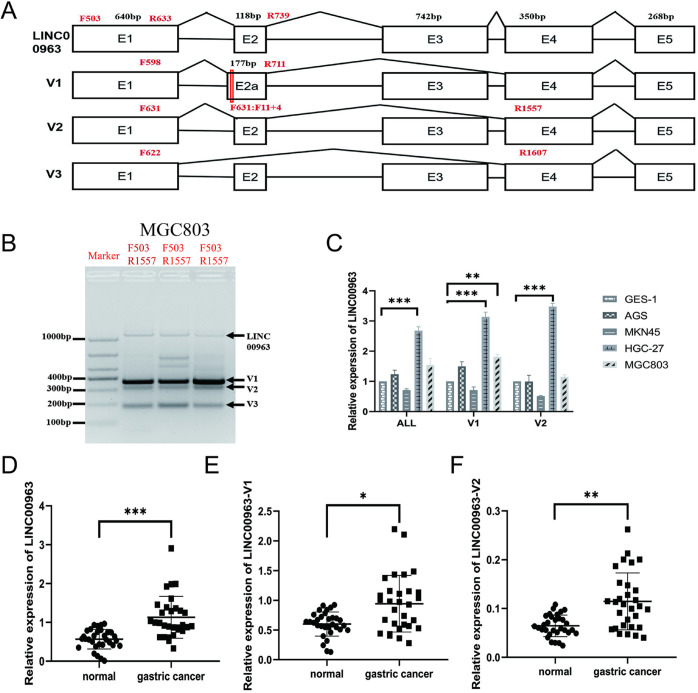
LINC00963 discovery of new alternative isoforms and expression analysis. (A): *LINC00963* the structure of the newly discovered splice variants, the number of exons and bases and introns, and the binding sites of all primers, the siRNA design is located within the red box. (B): The theoretical length of *LINC00963*-F503/R1557 is 1054bp. Three unreported alternative isoforms of *LINC00963* gene were isolated, sequenced and analyzed by 2% agarose gel in gastric cancer cell line MGC803. The 5 ’end of the second exon of V1 is 59 more bases than the second exon of wild type transcript (NR 038955.1). (C): The relative expression analysis of the total alternative isoforms (ALL) of the *LINC00963* gene and the newly discovered alternative isoforms in gastric cancer cell lines with the GES-1 cell line as a control. (D): The expression of all alternative isoforms of the LINC00963 gene in the peripheral blood of gastric cancer patients and healthy individuals at the time of a physical examination. (E): The expression of *LINC00963*-V1 in the peripheral blood of gastric cancer patients and healthy individuals at the time of a physical examination. (F): The expression of *LINC00963*-V2 in the peripheral blood of gastric cancer patients and healthy individuals at the time of physical examination. *P < 0.05, **P < 0.01, ***P < 0.001.

The specific primers were designed to amplify *LINC00963* gene expression and its alternative splice variants. It was shown that through cloning and sequencing, the gene had multiple alternative splice variants that were not included in the NCBI *LINC00963*-V1(Banklt2765306 OR820605), *LINC00963*-V2(Banklt2765288 OR820604), and *LINC00963*-V3(Banklt2765324 OR820606) (Fig 3A–3B). Sequencing results revealed the appearance of V1 with an additional 59 bases at the 5 ’end of the second exon (Fig 3A). This addition was entirely consistent with the 59 bases at the 3’ end of the first intron, and the third exon was completely deleted. Variant 2 skips the third exon, while variant 3 skips both the second and third exons. The expression of *LINC00963* and its newly discovered alternative splice variants were explored in gastric mucosal (GES-1) and GC cell lines (AGS, MKN45, HGC-27, and MGC803). In comparison to the GC HGC-27 cell line, *LINC00963* gene expression was over tripled than the GES-1 cell line (Fig 3C). The level of *LINC00963*-V1 expression in the MGC803 cell line was not as high as the HGC-27 cell line, but was higher than gastric mucosal GES-1 cells (Fig 3C). The peripheral blood of 30 patients with GC and the peripheral blood of 30 healthy individuals at the time of physical examinations were collected to investigate the expression *LINC00963*, revealing that in the peripheral blood of GC patients, the expression was higher than in the peripheral blood of healthy individuals (Fig 3D–3F). These results indicate that our newly discovered alternative splicing variants of *LINC00963* are highly expressed in gastric cancer and may play important biological functions.

*LINC00963 was associated with the promotion of EMT in GC cell lines, potentially through modulation of the Wnt/*β*-catenin signaling pathway.*

**Fig 4:**
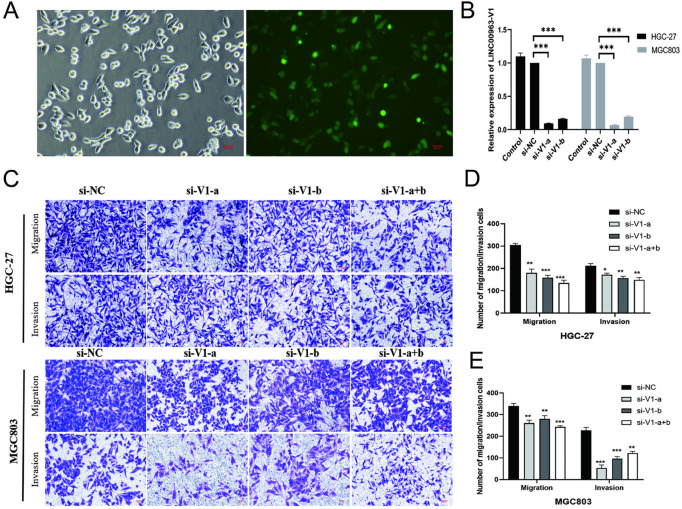
LINC00963-V1 knockdown to detect the invasion and migration ability of HGC-27 and MGC803 cells. (A–B): siRNA with FAM fluorophore was transfected into the cells to detect the cell transfection efficiency at 6 h. The RNA was extracted 48 h after transfection to detect the knockdown efficiency; the knockdown efficiency was >80%. (C–E): 48 h after transfection of cell line, detection of HGC-27 and MGC803 cell invasion and migration, showed that the ability of cell invasion and migration decreased after *LINC00963*-V1 knockdown compared with the si-NC group. *P < 0.05, **P < 0.01, ***P < 0.001.

The relative expression of *LINC00963*-V1 was highest among the alternative splice variants of the *LINC00963* gene (Fig 3B). The previous bioinformatics analysis showed that super-enhancer-associated genes were involved in cell metastasis (Fig 2B). The migration and invasion ability of HGC-27 and MGC803 cell lines were significantly weakened after knockdown of the *LINC00963*-V1 group compared to the si-NC group (Fig 4A–4E).

**Fig 5:**
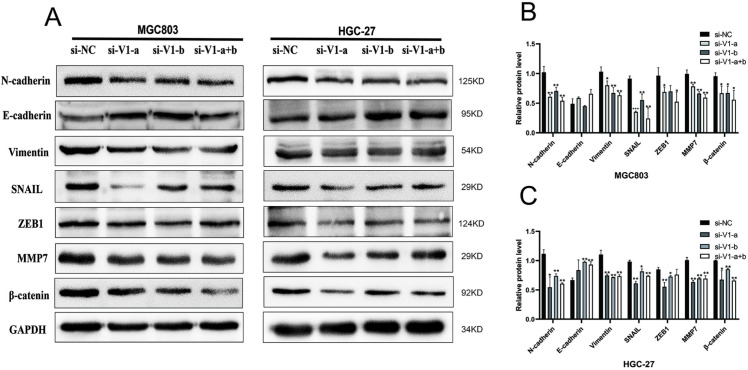
LINC00963-V1 knockdown attenuated EMT in GC HGC-27 and MGC803 cells. (A–C): The EMT-associated proteins in HGC-27 and MGC803 cells were detected by western blot assay after si-V1-a, si-V1-b, and si-NC transfection compared with the si-NC group. *P < 0.05, **P < 0.01, ***P < 0.001.

The EMT process is crucial for gastric cancer cell metastasis. Research has shown that knockdown *LINC00963* in the *LINC00963*-V1 group leads to increased expression of epithelial marker *E-cadherin* and decreased expression of mesenchymal markers *N-cadherin* and *vimentin* compared to the si-NC group. Moreover, expression of the EMT-related transcription factors *SNAIL, ZEB1* and *β*-*catenin* were downregulated upon knockdown of *LINC00963*, indicating that high expression of *LINC0963* promotes the occurrence and development of EMT (Fig 5A–5C). The results of WB three replicates are detailed in S1 Fig and S2 Fig.

*Suppression of LINC00963 inhibited GC cells growth in vivo*

**Fig 6:**
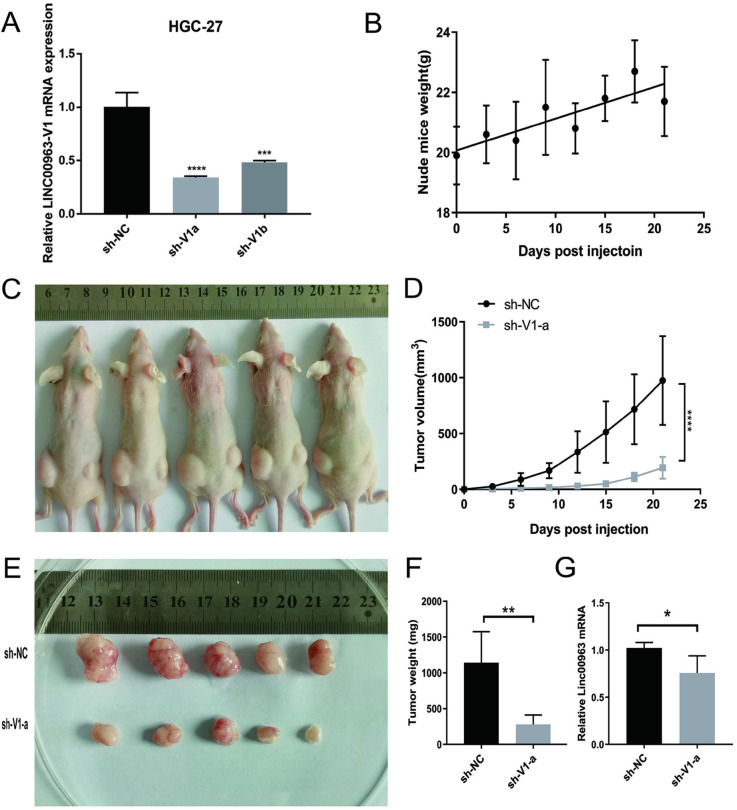
Stable knockdown of LINC00963 expression inhibited the invasion, migration, and tumor growth of the gastric cancer cell line, HGC-27. (A). Lentivirus vector plasmid stably knockdown of *LINC00963* expression. (B–D). The tumorigenesis experiment, B) Average weight of nude mice, C) Photo of tumor volume in vivo, the left side was sh-NC, and the right side was sh-*LINC00963*-V1-a. (D). The curves of tumor volumes. (E–F). Pictures and weight statistics of tumors in vitro. (G). The expression of *LINC00963* in tumor tissue. *P < 0.05, **P < 0.01, ***P < 0.001.

In order to investigate the effect of *LINC00963* on tumorigenicity in vivo, we inoculated sh-LINC00963 group tumor cells into the right groin of mice, and sh-NC group tumor cells into the left groin of BALB/c mice, establishing a mouse subcutaneous xenograft model. After 21 days of observation and feeding, euthanize the mice to remove the transplanted tumor. Fig 6A demonstrates that the knockdown efficiency of sh-LINC00963-V1-a was higher than that of sh-LINC00963-V1-b. Throughout the experimental period, nude mice exhibited a steady increase in body weight, as depicted in Fig 6B. Cells transfected with lenti-shLINC00963 V1 displayed tumor volume was significantly reduced and weight, as illustrated in Fig 6C and 6D. After euthanizing the mice, the results showed that the tumor weight of the *LINC00963* knockdown group was significantly smaller than that of the control group, indicating that knockdown the expression of *LINC00963* significantly inhibited tumor growth. As shown in the figure (Fig 6E, 6F). Then, RNA was extracted from the tumor tissue for detection, and it was found that the expression of *LINC00963* in the sh-LINC00963 side of the tumor tissue was lower than that in the control side, as shown in Fig 6G.

## Discussion

As an important cis-acting element, super-enhancers are rich in transcription factors and cofactors, such as high-density *BRD4*, which greatly enhance gene expression [21–23]. Accumulating studies indicate that super-enhancers play a pivotal role in regulating target genes, fostering the malignant progression of tumors [24–27] The *LINC00963* gene is situated on human chromosome 9q34.11, and research indicates that its disorders are commonly involved in tumor invasion, metastasis, and disease deterioration [28–30]. However, it remained unclear whether *LINC00963* is associated with any super enhancers. In our study, we comprehensively characterized the super-enhancer landscape in GC by analyzing uploaded H3K27ac ChIP-seq data from GC cell lines (GSE75595). Subsequently, we identified *LINC00963* as a super-enhancer-associated gene. It has been reported that transcripts sensitive to JQ1 are potential super-enhancer associated genes [31]. Our results demonstrated that low doses of JQ1 inhibited the transcription of *LINC00963* in GC cell lines. These findings indirectly suggest that *LINC00963* is regulated by super-enhancers. Furthermore, we observed high expression of *LINC00963* in both GC cell lines and the peripheral blood of GC patients, implying a potential role for *LINC00963* in the initiation and progression of GC.

The types of proteins in humans have been shown to be far greater than the number of genes, due in part to multiple alternative splicing in genes translate proteins with different functions. We found multiple alternative splicing variants in GC, which may have a crucial role in the malignant progression of tumors. Our results showed that *LINC00963* has at least three variants not included in the NCBI database, while the level of wild-type transcript (NR 038955.1) expression was very low. In several studies involving the alternative splicing of the *LINC00963* gene, the newly discovered splicing variants is the deletion of the 5’ end sequence [32,33]. Our research results showed that the *LINC00963* alternative splicing was mainly caused by the 59 base increase in the 5’ end of the second exon and the deletion of the third exon, which may be due to the splicing variants produced by different malignant tumors. Notably, we did not perform a 5’ RACE test to verify whether there is a deletion of the RNA 5’ end of the *LINC00963* gene. Because there are multiple splicing variants in *LINC00963* and the *LINC00963*-V1 expression was the highest, it is still unknown whether the splice variants event of *LINC00963* was related to the mutation or deletion of some sites of *LINC00963*, which affected the post-transcriptional modification of RNA, this will be the focus of our further research. The *LINC00963* expression was downregulated in both AGS and MKN45 cells. Although bioinformatics analysis showed that *LINC00963* was the target gene of the super-enhancer in AGS and MKN45 cell lines, the reason for this result might be the high degree of differentiation between AGS and MKN45. The differences caused by the different sources of cancer cells when the cells were constructed may also be caused by some mutations in the continuous culture of cell lines, which may result in the low expression of *LINC00963*or the weakened function of super-enhancers. In conclusion, the high expression of *LINC00963* and its different splicing variants in peripheral blood indicates that *LINC00963* may be a potential molecular marker of GC, which implys a new insight for *LINC00963* studies.

At present, accumulated studies have shown that EMT is closely related to the invasion and metastasis of tumors, and involves the signal transmission of Wnt/*β*-catenin, TGF-*β*, and other related pathways [34–36]. After the knockout of *LINC00963*-V1 in vitro, the invasion and migration ability of GC cells were greatly weakened, the EMT-related proteins changed, and the expression of *β*-catenin was decreased. As *β*-catenin is an important molecular marker in the Wnt/*β*-catenin signaling pathway, the Wnt/*β*-catenin signaling pathway has been associated with EMT in multiple tumor studies [17,37]. Numerous studies have shown that *LINC00963* has an oncogene role in tumor occurrence and development, promoting the metastasis and invasion of cancer cells [38–42], which is consistent with our results. Our research results indicate that *LINC00963* is a spliced long non-coding RNA regulated by super-enhancers, which is associated with EMT-mediated tumor progression. Future studies are needed to dissect the mechanism of isoform specificity. *LINC00963* may promote epithelial-mesenchymal transition (EMT) in gastric cancer (GC) cells by participating in the Wnt/*β*-catenin signaling pathway, thereby enhancing their invasion and migration abilities. It is noteworthy that we observed variations in the phenotypic intensity induced by *LINC00963* knockdown across different cell lines. For instance, the upregulation of E-cadherin was more pronounced in MGC803 cells compared to HGC-27 cells. We speculate that this cell-dependent nature may stem from differences in their intrinsic EMT status and genetic background. Reports have indicated that HGC-27 cells carry *TP53* mutations [43], which may make them more reliant on other EMT drivers (such as *TWIST1*), thereby reducing their dependence on *LINC00963*. An intriguing finding is that the combined use of two siRNAs (si-V1a+b) did not yield a stronger additive effect compared to a single siRNA. This suggests that the regulation of E-cadherin may exhibit a nonlinear dose-response relationship and a saturation phenomenon. That is, partial knockdown of *LINC00963* may already be sufficient to relieve its maximum inhibition on E-cadherin, reaching a plateau, where further knockdown fails to induce stronger phenotypic changes. Surprisingly, in some cases, single si-V1a induced stronger phenotypic changes compared to the mixed siRNA. This may be due to competition among different siRNAs in terms of RNA-induced silencing complex (RISC) loading efficiency, or interference from their unique off-target effects. The high potency of si-V1a also hints that specific subtypes of *LINC00963-V1* may play a more pivotal role. This discovery warrants further verification through subtype-specific knockout using gene editing technologies such as CRISPR/Cas9 in the future, to circumvent potential limitations of siRNA.

However, the mechanisms underlying the invasion and metastasis of gastric cancer (GC) involve numerous cellular regulatory processes. *LINC00963* may merely represent a subset of tumor metastasis. This study demonstrates that *LINC00963* splice variants are highly expressed to varying extents in the peripheral blood of gastric cancer patients. Compared to normal peripheral blood, these variants may originate from leukocytes, circulate freely in plasma, or enter the blood circulation as gastric cancer cells. However, the specific source remains unclear, which will be the focus of our next step of work. Research indicates that JQ1 can sensitively interfere with the function of super-enhancers, but it cannot definitively interfere with the expression of specific genes. Our experimental results also indicate that *LINC00963* is a long non-coding RNA regulated by super enhancers and spliced, which is involved in EMT mediated tumor progression. At the same time, studying splicing isoforms in vivo is of great value and represents the direction of future work. We will further design experiments to verify the biological functions of different alternative isoforms of *LINC00963* and whether they are directly regulated by super enhancers.

## Conclusion

In summary, this study unveiled multiple previously unreported alternative splicing variants of *LINC00963* and explored its potential involvement in tumor invasion and migration, possibly through mechanisms linked to super-enhancer activity. These findings offered new insights into *LINC00963* as a potential biomarker for GC metastasis and a promising therapeutic target in gastric cancer treatment.

## Supporting information

S1 Fig S1_raw_images. HGC-27 original results were repeated 3 times – blot.
(TIF)

S2 Fig S2_raw_images. MGC-803 original results were repeated 3 times – blot.
(TIF)

S3 Appendix Supplementary Material. (DOCX)

S4 Appendix Minimal data set. (ZIP)
