# Supplementary figures and images for: Super-enhancer-associated LINC00963 promotes metastasis of gastric cancer through epithelial-mesenchymal transition
Source: PLoS One. 2025 Sep 18;20(9):e0332396. doi: 10.1371/journal.pone.0332396 (PMC12445500; doi:10.1371/journal.pone.0332396)

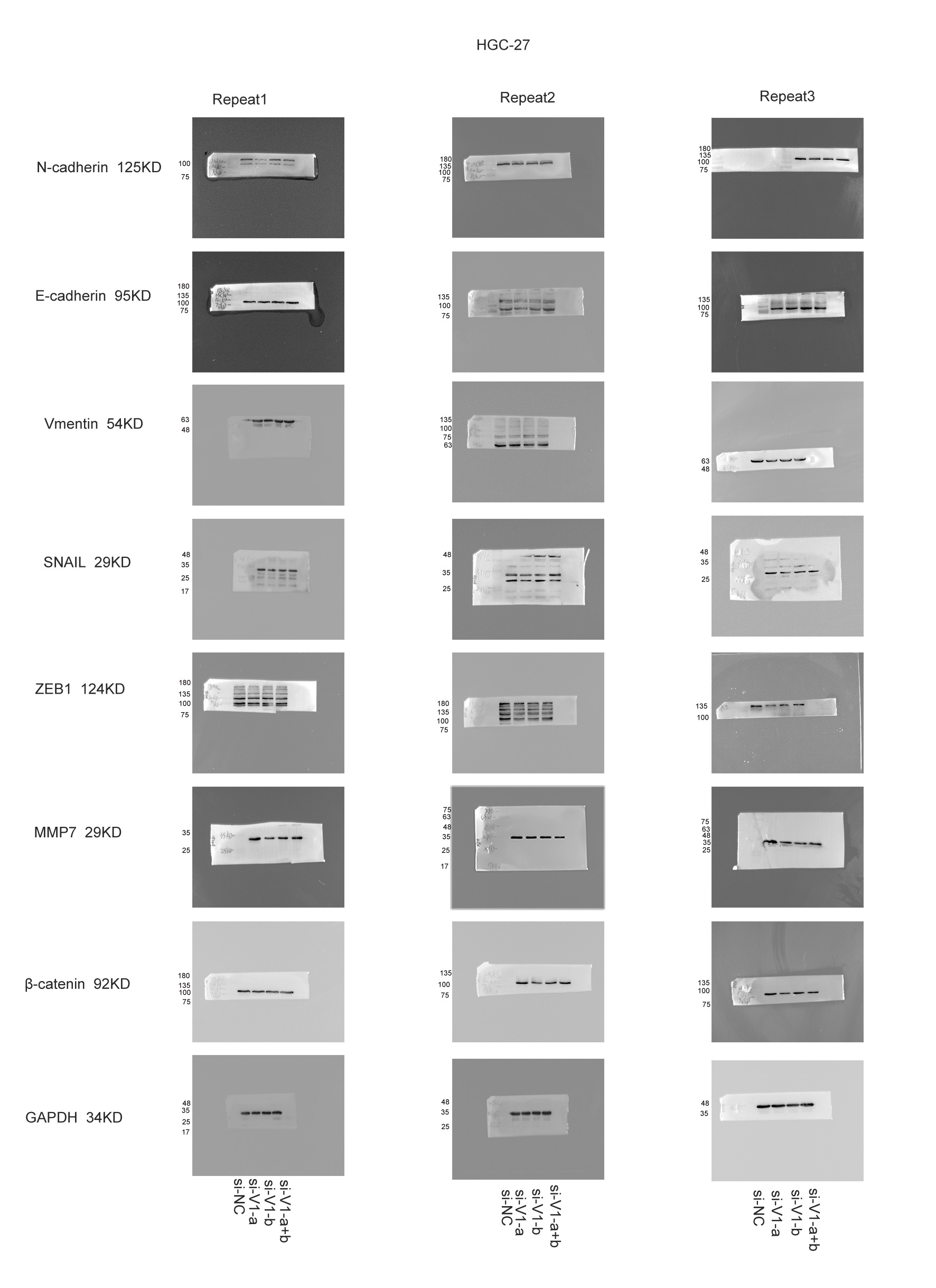

Supplement: S1 Fig — HGC-27 original results were repeated 3 times – blot. (TIF) [file pone.0332396.s001.tif]

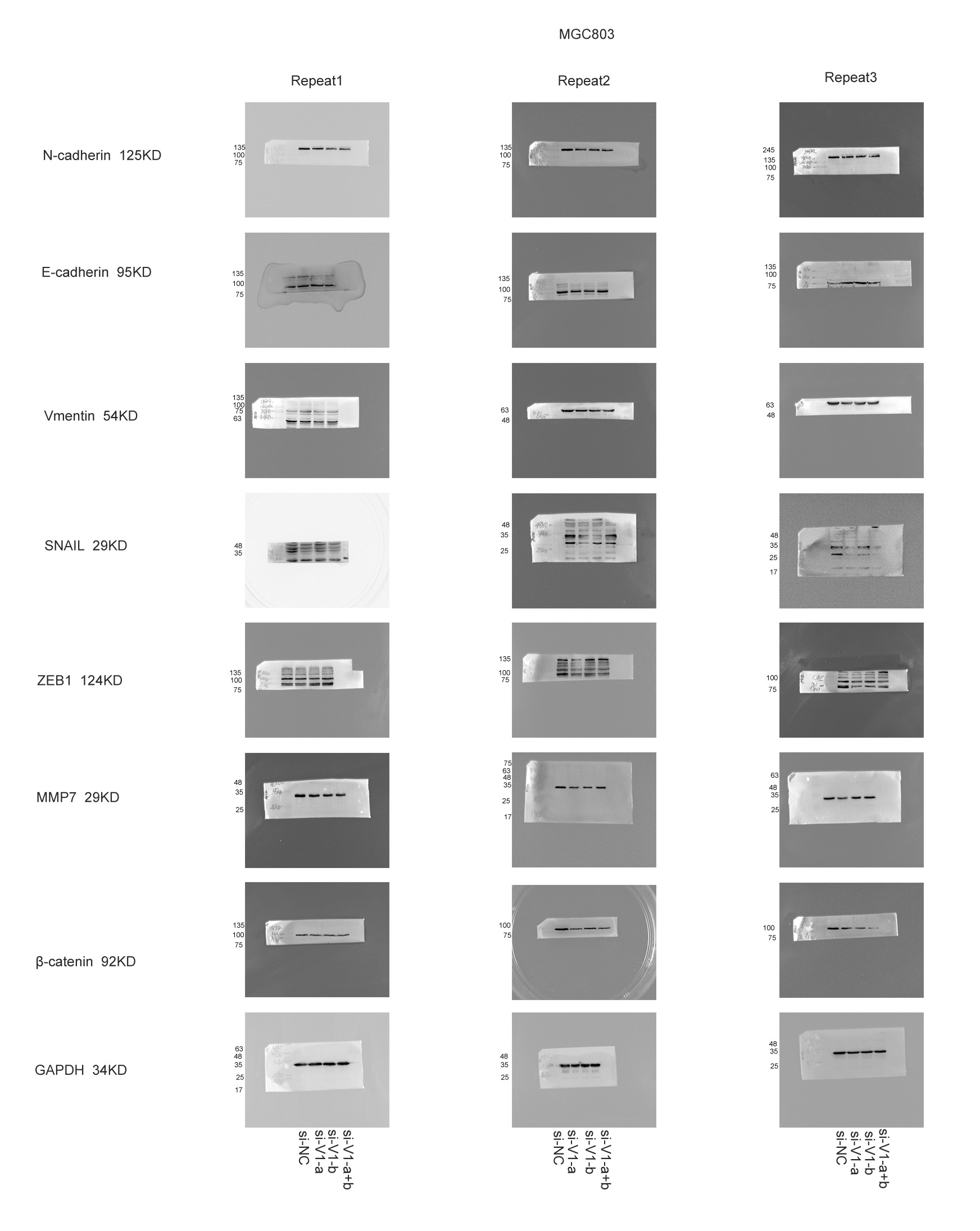

Supplement: S2 Fig — MGC-803 original results were repeated 3 times – blot. (TIF) [file pone.0332396.s002.tif]
