# Supplementary material for: Super-enhancer-associated LINC00963 promotes metastasis of gastric cancer through epithelial-mesenchymal transition
Source: PLoS One. 2025 Sep 18;20(9):e0332396. doi: 10.1371/journal.pone.0332396 (PMC12445500; doi:10.1371/journal.pone.0332396)
Supplement: S3 Appendix — (DOCX) [file pone.0332396.s003.docx]

Supplementary Material

**Super-enhancer-associated LINC00963 promotes metastasis of gastric cancer through epithelial-mesenchymal transition**

# Supplementary Tables

**S1 Table.** Primers

| **Name** | **Sequences** |
| --- | --- |
| HPRT-F148 | 5’-ATGGCGACCCGCAGCCCT-3’ |
| HPRT-R266 | 5’- CCATGAGGAATAAACACCCT-3’ |
| LINC00963-F687（NR_038955.1） | 5 '- CTGTGCCAGACACTGAAGAT-3' |
| LINC00963-R807（NR_038955.1） | 5'- GAGATCACACCACTGTACT-3' |
| LINC00963-F503（ALL） | 5'- GGTCAGGCCACTCTGCTACT-3' |
| LINC00963-R633（ALL） | 5'- ATGGGCTTCCAACTGCGATG-3' |
| LINC00963-F598（V1） | 5'- CAAAGGAGAGCACAACCAT-3' |
| LINC00963-R711（V1） | 5'-ATCTGCACTGGGCTTGAAAT-3' |
| LINC00963-F631（V2） | 5'- CATGGGGCAGCCCAG-3' |
| LINC00963-R1557（V2） | 5'-CATGCCTGTAAGTAGCCACT-3' |

LINC00963: long intergenic non-protein coding RNA 963; HPRT: hypoxanthine phosphoribosyltransferase 1; F: forward; R: reverse. ALL: all variant; V1: variant 1; V2: variant 2.

**S2 Table.** siRNA

| **Name** | **Sequences** |
| --- | --- |
| si-V1-a | 5'- GACACAGUGAGCAUACAAUTT-3' |
| si-V1-b | 5'-CUGUGGUGUCUUGAUUUCATT-3' |
| si-NC | 5’-UUCUCCGAACGUGUCACGUTT-3’ |
| LINC00963-V1 has an additional 59 base sequences at the 5 'end of the second exon, with the underlined portion indicating the specific location of the siRNA | 5’-GGCCCAGGACACAGTGAGCATACAAT  AGACATTAGCTGCTGTGGTGTCTTGATTTCA  AG-3’ |

NC, negative control; si, small interfering; si-V1-a: small interfering – variant 1-a; si-V1-b: small interfering – variant 1-b; LINC00963: long intergenic non-protein coding RNA 963.

**S3 Table.** shRNA

| **Name** | **Sequences** |
| --- | --- |
| sh-V1-a | 5'- GACACAGTGAGCATACAATTTAAATTGTATGCTCACTCTCTC-3' |
| sh-V1-b | 5'-CTGTGGTGTCTTGATTTCATTAATGAAATCAAGACACCACAG-3' |
| sh-NC | 5’-TTCTCCGAACGTGTCACGTTTAAACGTGACACGTTCGGAGAA -3’ |

NC, negative control; sh, short hairpin; sh-V1-a: short hairpin-variant 1-a; sh-V1-b: short hairpin- variant 1-b.
